# Kaiso depletion attenuates transforming growth factor-β signaling and metastatic activity of triple-negative breast cancer cells

**DOI:** 10.1038/oncsis.2016.17

**Published:** 2016-03-21

**Authors:** B I Bassey-Archibong, J M Kwiecien, S B Milosavljevic, R M Hallett, L G A Rayner, M J Erb, C J Crawford-Brown, K B Stephenson, P-A Bédard, J A Hassell, J M Daniel

**Affiliations:** 1 Department of Biology, McMaster University, Hamilton, Ontario, Canada; 2 Department of Pathology and Molecular Medicine, McMaster University, Hamilton, Ontario, Canada; 3 Department of Neurosurgery and Paediatric Neurosurgery, Medical University of Lublin, Lublin, Poland; 4 Department of Biochemistry and Biomedical Sciences, McMaster University, Hamilton, Ontario, Canada; 5 Department of Psychology, Neuroscience and Behaviour, McMaster University, Hamilton, Ontario, Canada; 6 Centre for Innovative Cancer Research, Ottawa Hospital Research Institute, Ottawa, Ontario, Canada

## Abstract

Triple-negative breast cancers (TNBCs) represent a subset of breast tumors that are highly aggressive and metastatic, and are responsible for a disproportionate number of breast cancer-related deaths. Several studies have postulated a role for the epithelial-to-mesenchymal transition (EMT) program in the increased aggressiveness and metastatic propensity of TNBCs. Although EMT is essential for early vertebrate development and wound healing, it is frequently co-opted by cancer cells during tumorigenesis. One prominent signaling pathway involved in EMT is the transforming growth factor-β (TGFβ) pathway. In this study, we report that the novel POZ-ZF transcription factor Kaiso is highly expressed in TNBCs and correlates with a shorter metastasis-free survival. Notably, Kaiso expression is induced by the TGFβ pathway and silencing Kaiso expression in the highly invasive breast cancer cell lines, MDA-MB-231 (hereafter MDA-231) and Hs578T, attenuated the expression of several EMT-associated proteins (Vimentin, Slug and ZEB1), abrogated TGFβ signaling and TGFβ-dependent EMT. Moreover, Kaiso depletion attenuated the metastasis of TNBC cells (MDA-231 and Hs578T) in a mouse model. Although high Kaiso and high TGFβR1 expression is associated with poor overall survival in breast cancer patients, overexpression of a kinase-active TGFβR1 in the Kaiso-depleted cells was insufficient to restore the metastatic potential of these cells, suggesting that Kaiso is a key downstream component of TGFβ-mediated pro-metastatic responses. Collectively, these findings suggest a critical role for Kaiso in TGFβ signaling and the metastasis of TNBCs.

## Introduction

Breast cancer is the most common female cancer and a leading cause of female deaths worldwide.^1^ Of the five major breast cancer subtypes,^2, 3^ the triple-negative breast cancers (TNBCs) have the worst prognosis because of their limited treatment options and highly metastatic nature.^4, 5^ Several studies suggest a role for the epithelial-to-mesenchymal transition (EMT) program in the metastatic propensity of TNBCs.^6, 7^ Indeed, increased expression of various EMT proteins (for example, Vimentin, ZEB1) has been reported in many TNBC cases, where they appear to correlate with increased invasiveness and poor disease-free survival.^7, 8^

EMT is a complex and tightly regulated process that confers mesenchymal properties (for example, increased motility and invasiveness) to epithelial cells (reviewed in Kalluri and Weinberg^9^). The switch in cellular behavior and characteristics during EMT is accomplished mostly by EMT-associated transcription factors (for example, Snail/Slug, ZEB1/2) that function to promote the loss of epithelial components (for example, E-cadherin) and gain of mesenchymal proteins (for example, Vimentin). These EMT transcription factors are activated by many cytokines or growth factors including the transforming growth factor-β (TGFβ) (reviewed in Puisieux *et al.*^10^).

The TGFβ pathway controls many normal and pathological processes in addition to EMT.^11, 12^ TGFβ signals are transduced either via the canonical cascade involving Smad proteins (for example, Smad2/3) or the noncanonical cascade involving non-Smad proteins (for example, phosphatidylinositol 3 kinase/AKT, extracellular signal-regulated protein kinase-1/2; reviewed in Zhang^12^ and Heldin *et al.*^13^). Depending on the cellular context, TGFβ suppresses or promotes tumor progression in breast cancers (BCa). In early-stage BCa, TGFβ is a potent inhibitor of uncontrolled cell proliferation; however, in advanced BCa, TGFβ promotes metastasis as the cells become refractory to TGFβ growth inhibition.^14^ The mechanism underlying the switch in TGFβ function from a tumor suppressor to tumor promoter is not well understood but studies implicate the TGFβ receptors (TGFβR1 and 2) as critical determinants of the functional specificity of the TGFβ signaling cascade.^15, 16, 17^ A metastasis-associated TGFβ response signature that includes expression of several EMT-associated genes was recently identified in breast tumors,^18^ further highlighting the importance of TGFβ signaling in EMT induction and malignant progression of BCa.

Recently, the transcription factor Kaiso was identified as a regulator of E-cadherin expression and EMT in prostate and breast tumors.^19, 20^ Kaiso is a unique dual-specificity transcription factor that recognizes and binds a consensus Kaiso-binding sequence (KBS), TCCTGCNA, or methylated CpG-dinucleotides.^21^ Most Kaiso target genes (for example, *CCND1*, *S100A4*, *MMP7*, *CDH1*) identified to date are linked to tumor onset, progression and metastasis.^22, 23, 24, 25^ Thus, not surprisingly, Kaiso is implicated in various human cancers (breast, colon, lung, prostate), and appears to have both tumor suppressive and promoting roles.^19, 20, 26, 27, 28, 29, 30^ Indeed, high Kaiso expression correlates significantly with estrogen receptor-α negativity, basal/TNBCs and poor prognosis in patients with infiltrating BCa.^20, 29^ More recently, Kaiso was implicated as a potential drug target in glucocorticoid-combined chemotherapy in breast cancer.^30^ However, the precise roles and mechanism of action of Kaiso in tumorigenesis remain poorly understood. Here, we report that high Kaiso expression in BCa patients correlates with high expression of the TGFβ signalsome and shorter metastasis-free survival. Silencing Kaiso expression in TNBC cells attenuates TGFβ signaling and TGFβR1 expression, and induces an EMT reversal concomitant with decreased EMT protein expression. More importantly, silencing Kaiso strongly inhibited TNBC cell metastasis in two mouse metastasis models. However, although expression of a constitutively active TGFβR1 in Kaiso-depleted TNBC cells rescued TGFβ signaling, this was insufficient to restore the metastatic abilities of these cells. Our results present the first evidence linking Kaiso to TGFβ signaling and BCa metastasis *in vivo*, and highlight a clinically relevant role for Kaiso in the metastasis of aggressive breast tumors.

## Results

### High Kaiso expression correlates with poor prognosis in breast cancer patients

**Figure 1:**
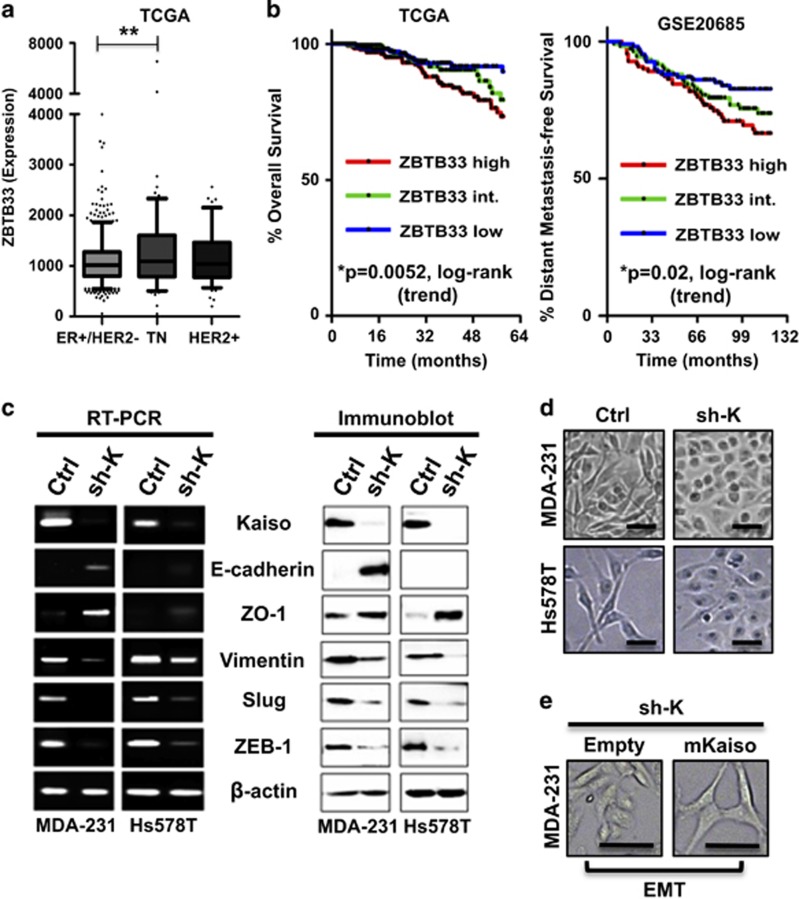
High Kaiso expression correlates with shorter metastasis-free survival and EMT. (**a**) Analysis of the publicly available TCGA breast cancer (BCa) data set revealed that high Kaiso expression correlates with ER (−) negativity and TNBC. **P<0.001. (**b**) Patients from the TCGA (*n*=977) and the GEO (GSE20685) (*n*=327) data sets were segregated into Kaiso (ZBTB33)-high, Kaiso-intermediate and Kaiso-low groups based on transcript levels. Kaplan–Meier survival curves revealed a significant negative correlation between high Kaiso expression, overall survival and distant metastasis-free survival in all BCa cases. Statistical significance was determined by log-rank test and *P*-values are indicated. (**c**) RT–PCR and immunoblot analysis of control and Kaiso-depleted MDA-231 and Hs578T cells. (**d**) Phase-contrast images of control and Kaiso-depleted MDA-231 and Hs578T cells. (**e**) Phase-contrast images of Kaiso-depleted MDA-231 cells transfected with either an empty or mKaiso vector. Scale bar, 100 μM.

Kaiso is highly expressed in several TNBC cell lines (our unpublished data) and nuclear Kaiso expression has been linked with EMT and TNBC aggressiveness.^20, 29^ To determine the clinical relevance of Kaiso (ZBTB33) expression in aggressive BCa, we analyzed The Cancer Genome Atlas (TCGA) and the Gene Expression Omnibus (GEO) (GSE20685) breast cancer data sets. Consistent with an earlier study,^29^ most high Kaiso-expressing tumors lacked the estrogen receptor. However, the highest and most statistically significant Kaiso expression correlated with TNBC cases (Figure 1a). Importantly, Kaplan–Meier survival curves revealed that patients with high Kaiso-expressing tumors (ZBTB33 high) had a poorer overall survival (log-rank test, *P*=0.0052) and shorter distant metastasis-free survival (log-rank test, *P*=0.02) compared with patients with intermediate or low Kaiso-expressing tumors (Figure 1b) in all BCa cases. These findings suggested a clinically relevant role for Kaiso in TNBC.

### Kaiso-depleted TNBC cell lines undergo mesenchymal-to-epithelial transition

As a first step to unraveling the function of Kaiso in TNBC, we generated stable Kaiso depletion in two highly invasive TNBC cell lines (MDA-231 and Hs578T) using two independent Kaiso-specific short hairpin (sh)-RNAs. As Kaiso was linked to EMT,^20^ we first confirmed that Kaiso depletion (sh-K1, sh-K2) altered the expression of the EMT proteins E-cadherin and Vimentin (Figure 1c and Supplementary Figure 1). Increased E-cadherin expression was observed in Kaiso-depleted (sh-K) MDA-231 cells but not in Hs578T counterparts (Figure 1c and Supplementary Figure 1). In contrast, Kaiso-depleted MDA-231 and Hs578T cells both exhibited decreased expression of the EMT-inducing transcription factors Slug and ZEB1 but increased expression of the epithelial protein ZO-1 (Figure 1c). These gene expression changes resulted in a concomitant induction of a mesenchymal-to-epithelial transition phenotype in Kaiso-depleted MDA-231 and Hs578T cells (despite Hs578T-sh-K cells lacking any obvious E-cadherin expression) (Figure 1d). Re-expression of a sh-resistant Kaiso complementary DNA (cDNA; mKaiso) in MDA-231-sh-K cells restored the mesenchymal phenotype (Figure 1e). Thus, in addition to directly regulating E-cadherin expression,^19, 20^ Kaiso may indirectly regulate E-cadherin and EMT via modulation of transcription factors that repress E-cadherin.

### Kaiso depletion attenuates the metastasis of TNBC cells

**Figure 2:**
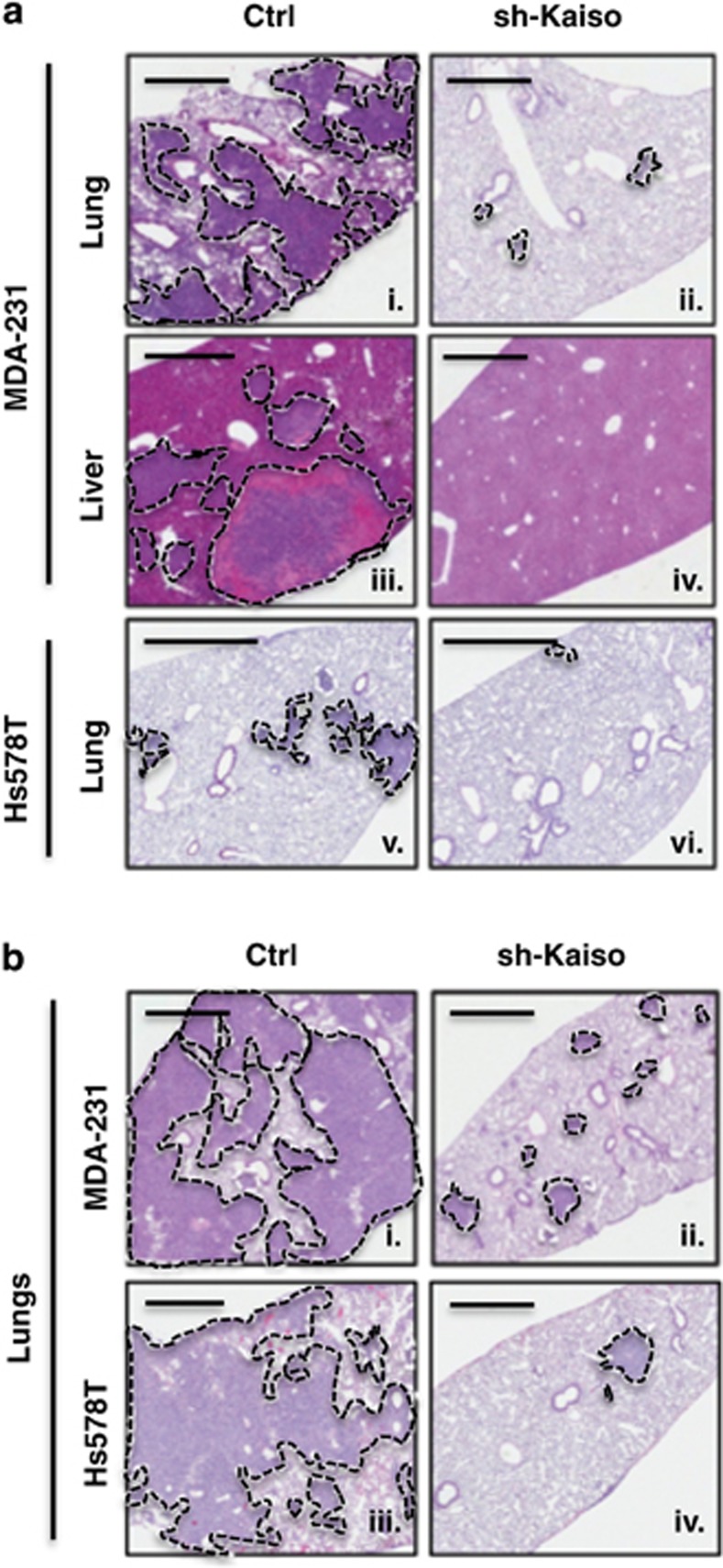
Kaiso depletion inhibits breast tumor cell metastasis to the lungs and liver. (**a** i–vi) Hematoxylin and eosin (H&E) staining of murine lungs and liver revealed that control MDA-231 xenografts formed extensive metastases in lungs (i) and liver (iii), whereas control Hs578T xenografts formed moderate metastases that were limited to the lungs (v) of immune-deficient mice. In contrast, Kaiso-depleted MDA-231 xenografts formed very few metastases in the lungs (ii), and no metastases in the liver (iv) of immune-deficient mice. Kaiso-depleted Hs578T xenografts also formed negligible metastases in the lung (v). (**b**) H&E images showing extensive metastases of control MDA-231 (i) and control Hs578T (iii) in the lungs of NSG mice after tail vein injections compared with few metastases formed by Kaiso-depleted MDA-231 (ii) and Hs578T cells (iv). Scale bar, 1000 μm. Representative images are shown.

The link of Kaiso to distant metastasis-free survival in BCa patients and EMT (Figure 1)^19, 20^ led us to question whether Kaiso was essential for TNBC dissemination. Thus, we investigated the effect of Kaiso depletion on TNBC cell metastasis in a mouse model where Kaiso-depleted MDA-231 and Hs578T cells were injected subcutaneously into the mammary fat pads of immuno-compromised mice and allowed to form tumors. In support of our hypothesis, we found that Kaiso-depleted MDA-231 cells exhibited only a few small metastatic foci in the lungs (Figure 2a ii), but no detectable metastases in the liver (Figure 2a iv) or other organs (data not shown) in all xenografted mice (*n*=9). In contrast, MDA-231 control-injected mice exhibited extensive metastases to the lungs and liver (*n*=6; Figure 2a i and -iii) as previously shown.^32, 33^ Similarly, control Hs578T cells exhibited modest metastases that were limited to the lungs of all xenografted mice (*n*=7; Figure 2a v) compared with Kaiso-depleted Hs578T injected mice that displayed very few metastatic foci in the lungs (*n*=7; Figure 2a vi). Similar results were obtained in experimental lung metastasis (tail vein injections) studies; control MDA-231 (*n*=5) and Hs578T (*n*=5) cells formed large and multiple foci in the lungs of all injected mice (Figure 2b i and iii), whereas Kaiso-depleted MDA-231 (*n*=5) and Hs578T cells (*n*=5) formed few foci in the lungs of injected mice (Figure 2b ii and iv). Collectively, these findings highlight for the first time the importance of Kaiso expression on the metastasis of TNBC cells.

### Kaiso expression positively correlates with TGFβ signaling protein expression

**Figure 3:**
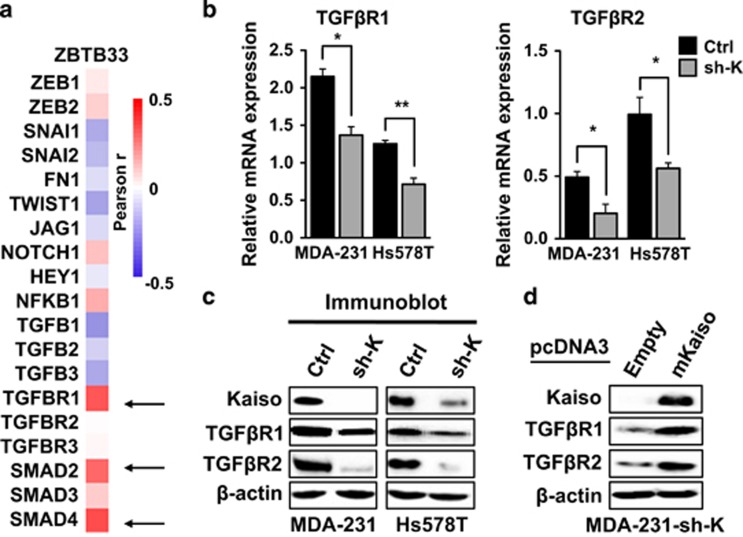
Kaiso expression positively correlates with TGFβ signaling components in triple-negative tumors. (**a**) Heat map showing the positive correlation between Kaiso expression and TGFβ signaling proteins. (**b**, **c**) Kaiso depletion attenuates TGFβR1 and TGFβR2 transcript and protein levels, as assessed by quantitative RT–PCR and immunoblot analysis, that is rescued upon re-expression of a sh-resistant Kaiso cDNA (**d**). β-Actin serves as a loading control. *P<0.05, **P<0.005.

To successfully undergo metastasis, tumor cells must activate various cellular processes in addition to EMT, to enable their extravasation, survival in the circulatory system and establishment at secondary sites.^34^ To elucidate how Kaiso might potentiate the complete metastatic cascade, we analyzed the TCGA BCa dataset to correlate Kaiso expression with other genes implicated in tumor progression and metastasis. We found that high Kaiso expression positively correlates with several TGFβ signaling genes including Smad2, Smad4 and TGFβR1 (Figure 3a). Examination of the expression levels of various TGFβ signaling components in Kaiso-depleted TNBC cells revealed that silencing Kaiso attenuated the expression of TGFβR1 and TGFβR2 at both the transcript and protein levels in both cell lines (Figures 3b and c). However, there were no significant changes in Smad2 or Smad4 expression in either cell line (data not shown). Notably, TGFβR1 and TGFβR2 expression was upregulated following expression of a sh-resistant Kaiso form in Kaiso-depleted MDA-231 cells (Figure 3d).

### Kaiso depletion attenuates TGFβ signaling and transcriptional responses

**Figure 4:**
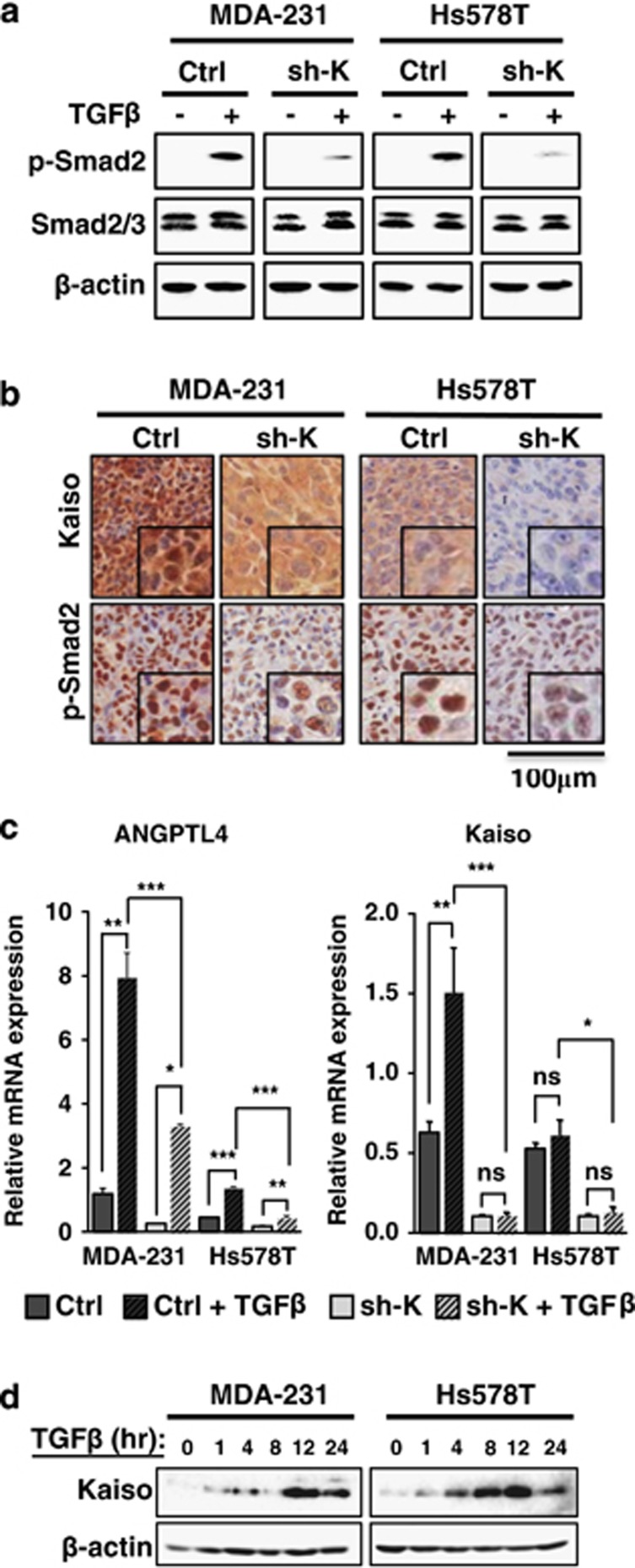
Kaiso-depletion attenuates TGFβ signaling and transcriptional responses. Cells were treated with 10 ng/ml of TGFβ for 1 h before assaying for TGFβ activity. (**a**) TGFβ treatment of control MDA-231 and Hs578T cells results in increased p-Smad2 levels. However, Kaiso-depleted MDA-231 and Hs578T cells treated with TGFβ display reduced p-Smad2 levels. (**b**) Kaiso-depleted MDA-231 and Hs578T xenografts exhibit decreased TGFβ signaling as evidenced by reduced p-Smad2 protein levels. (**c**) TGFβ-induced expression of *ANGPTL4* is attenuated in Kaiso-depleted cells treated with 10 ng/ml of TGFβ for 24 h. Interestingly, Kaiso expression is significantly increased by TGFβ treatment in MDA-231 cells. (**d**) Immunoblot analysis revealed a peak in Kaiso protein levels at 12 h in both MDA-231 and Hs578T cells in response to TGFβ treatment. All experiments were performed in triplicate. Representative images from all experiments are shown. *P<0.05, **P<0.005, ***P<0.0001, NS, not significant. β-Actin serves as a loading control.

The TGFβR1 and TGFβR2 serine/threonine kinases are essential for activation of the TGFβ signaling cascade.^14, 35, 36^ Hence, loss of either the expression or function of TGFβR1 or TGFβR2 perturbs TGFβ signaling.^37, 38, 39, 40^ As our Kaiso-depleted cells displayed decreased TGFβR1 and TGFβR2 expression, we hypothesized that suppressing Kaiso would attenuate TGFβ signaling. Indeed, Kaiso-depleted MDA-231 and Hs578T cells treated with recombinant human TGFβ1 had negligible levels of phosphorylated Smad2 (p-Smad2) that is indicative of active TGFβ signaling. This was in striking contrast to TGFβ1-treated MDA-231 and Hs578T control cells that exhibited increased p-Smad2 (Figure 4a). Consistent with our *in vitro* results, Kaiso-depleted MDA-231 and Hs578T mouse xenografts displayed reduced p-Smad2 expression *in vivo* compared with control MDA-231 and Hs578T xenografts (Figure 4b). To further validate the role of Kaiso in TGFβ-mediated signaling, we examined Kaiso-depletion effects on TGFβ-target gene expression. We chose *ANGPTL4* that is involved in TGFβ-mediated breast tumor cell homing to lungs^18^ as both control MDA-231 and Hs578T cells displayed a proclivity for lung metastasis. Silencing Kaiso significantly reduced TGFβ-induced expression of *ANGPTL4* (Figure 4c). Similarly, Kaiso depletion also attenuated TGFβ induction of *ZEB1* (Supplementary Figure 2) that participates in TGFβ-mediated EMT.^41^ Unexpectedly, we observed increased Kaiso (*ZBTB33*) transcript levels in response to TGFβ treatment in both cell lines (Supplementary Figure 3). This increase in Kaiso transcripts was abrogated by Kaiso-specific shRNA in Kaiso-depleted cells (Figure 4c). Persistent TGFβ treatment (1–24 h) also resulted in increased Kaiso protein levels that peaked at ~12 h in both cell lines (Figure 4d). Together, these results hint at a positive feedback loop between Kaiso expression and TGFβ signaling.

### Kaiso binds the TGFβR1 and TGFβR2 promoter endogenously

**Figure 5:**
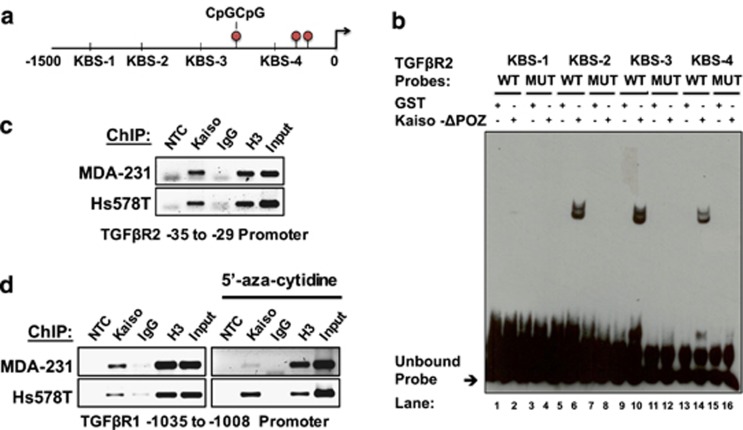
Kaiso associates with the endogenous TGFβR1 and TGFβR2 promoter in breast cancer cell lines. (**a**) Schematic illustration of the TGFβR2 promoter highlighting multiple KBS. Four double-stranded oligonucleotides were designed to contain core KBS from different regions of the promoter and utilized in electrophoretic mobility shift assay (EMSA) to determine Kaiso binding. (**b**) EMSA shows that Kaiso binds the proximal TGFβR2 promoter in a KBS-dependent manner (lanes 6, 10 and 14). This interaction was abolished (lanes 8, 12 and 16) upon introduction of a point mutation in the core KBS sequence of these probes or competition with the cold unlabeled wild-type probe. (**c**) ChIP of MDA-231 and Hs578T chromatin revealed that Kaiso binds the TGFβR2 promoter endogenously. (**d**) ChIP experiments of MDA-231 and Hs578T chromatin shows that Kaiso also interacts with the TGFβR1 promoter endogenously even after 5′-aza-cytidine treatment. Representative images are shown. All experiments were conducted in triplicate. H3, Histone 3 positive control; Input, 10% input. MUT, mutated; NTC, no template control; WT, wild type.

As Kaiso depletion attenuated TGFβR1 and TGFβR2 expression, we next assessed whether Kaiso promotes TGFβ signaling through regulation of TGFβR1 and TGFβR2. We performed electrophoretic mobility shift assay analyses using purified GST-Kaiso-ΔPOZ fusion proteins as previously described,^42, 43^ and oligonucleotides derived from the *TGFβR1* (KBS 1–4) and *TGFβR2* (KBS1–4) promoters that each contains several KBS and/or CpGs (Tables 1 and 2). Kaiso bound the core KBS in proximal TGFβR2 oligonucleotides (TβR2-KBS-2, 3, 4) but not the distal TβR2-KBS1 probe (Figures 5a and b). However, Kaiso binding was abolished upon introduction of a point mutation in the core KBS in these probes (Figure 5b; Supplementary Figure 4). Surprisingly, despite the strong correlation between Kaiso and TGFβR1 expression in the TCGA BCa dataset, no binding was observed between Kaiso and any of the TβR1-KBS-1–4 probes even after methylation of the CpG sequences found in the *TGFβR1* (KBS2-4) probes (data not shown).

Chromatin immunoprecipitation (ChIP) experiments subsequently revealed that Kaiso bound the endogenous *TGFβR2* promoter containing core KBS in MDA-231 and Hs578T cells (Figure 5c). Intriguingly, despite no direct interaction between Kaiso and the minimal *TGFβR1* promoter region *in vitro*, we found that Kaiso associated with the *TGFβR1* promoter endogenously (Figure 5d). As the amplified *TGFβR1* promoter region contained a CpG dinucleotide in addition to several core KBS (Table 1), we repeated the ChIP–PCR experiments using chromatin from MDA-231 and Hs578T cells treated with the demethylating agent 5′-aza-cytidine. Demethylation slightly abolished binding of Kaiso to the *TGFβR1* promoter in MDA-231 cells but had no effect on Kaiso binding in Hs578T cells (Figure 5d). The specificity of Kaiso binding to the *TGFβR1* and *TGFβR2* promoters was confirmed using primers designed against a distal region of both promoters lacking KBS or CpG sites (Supplementary Figure 5). Collectively, these results implicate both *TGFβR1* and *TGFβR2* as Kaiso target genes, and suggest that Kaiso may regulate *TGFβR1* expression indirectly, whereas it may directly regulate *TGFβR2* expression.

### High Kaiso and TGFβR1 expression correlates with poor survival in BCa patients

**Figure 6:**
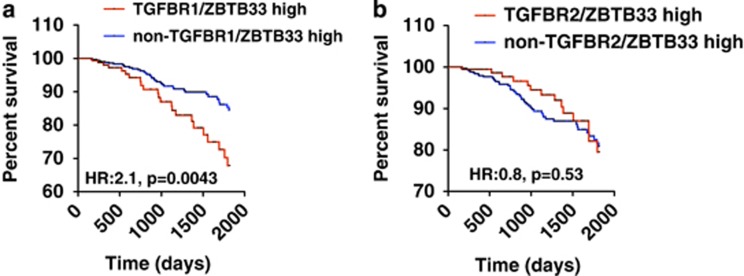
High Kaiso and TGFβR1 expression correlates with poor prognosis in breast cancer patients. (**a**) Kaplan–Meier survival curves show that high Kaiso and TGFβR1 expression correlates negatively with overall survival in the TCGA breast cancer data set. (**b**) High Kaiso and TGFβR2 expression does not correlate with overall survival in the TCGA breast cancer data set. Statistical significance was determined by log-rank test and *P-*values are indicated.

As the TGFβ pathway is highly implicated in BCa metastasis, we utilized the TCGA BCa dataset and correlated the expression levels of Kaiso, TGFβR1 or TGFβR2 with BCa survival. Consistent with Chen *et al.*,^44^ high TGFβR1 (Supplementary Figure 6) but not high TGFβR2 expression (data not shown) correlated with poor prognosis in BCa patients, although not significantly. Remarkably, increased Kaiso and TGFβR1 expression, but not increased Kaiso and TGFβR2 expression, correlated significantly with poor overall survival in BCa patients (Figures 6a and b). Kaiso may thus drive metastasis through TGFβR1 but not TGFβR2.

### Kinase-active TGFβR1 rescues TGFβ signaling but not the metastatic abilities of Kaiso-depleted MDA-231 cells

**Figure 7:**
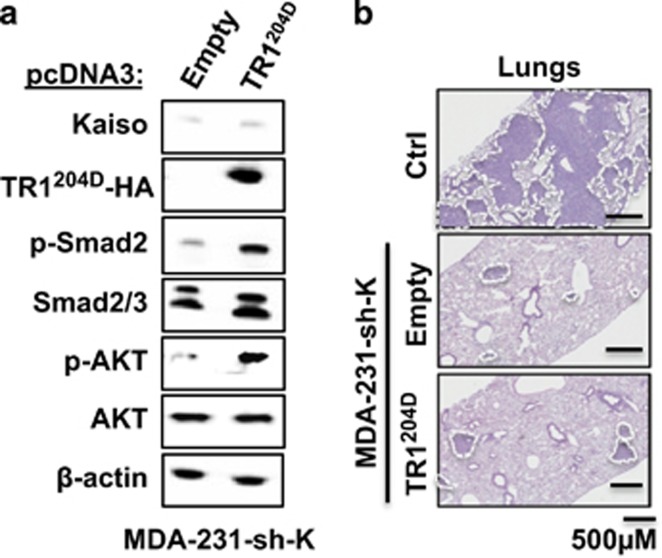
Re-expression of a constitutively active TGFβR1 in Kaiso-depleted cells is insufficient to restore breast cancer metastasis. (**a**) Overexpression of a constitutively active TGFβR1 (TR1^204D^) in Kaiso-depleted cells restores TGFβ signaling as evidenced by increased levels of p-Smad2 and p-Akt. (**b**) Hematoxylin and eosin (H&E) staining shows that overexpression of kinase-active TGFβR1 in Kaiso-depleted cells did not restore the metastatic capabilities of the cells. Representative images are shown. β-Actin serves as a loading control.

Based on the above findings, we questioned whether restoration of TGFβ signaling in Kaiso-depleted cells would restore their metastatic abilities. To address this, we overexpressed a constitutively kinase-active TGFβR1 (TRI^204D^) in Kaiso-depleted MDA-231 and Hs578T cells. TβRI^204D^ overexpression in Kaiso-depleted cells restored TGFβ signaling as evidenced by increased p-Smad2 and other non-Smad proteins (pAkt) compared with MDA-231-sh-K cells (Figure 7a). Remarkably, although TRI^204D^ overexpression restored TGFβ signaling, it was insufficient to restore the metastatic potential of the Kaiso-depleted cells (compare with metastatic foci generated by MDA-231-Ctrl cells in the lungs of injected mice) (Figure 7b). This suggested that Kaiso expression is important for TGFβ-mediated breast tumor metastasis.

## Discussion

Most cancer-related deaths are because of tumor metastasis to vital organs.^45^ The recent association of Kaiso with EMT^19, 20^ coupled with its misexpression in several aggressive cancers (prostate, breast) implicates Kaiso in metastasis. In this study we report for the first time that Kaiso depletion attenuated the metastatic ability of highly invasive TNBC cells (MDA-231 and Hs578T) in mouse models of metastasis. As our *in vitro* studies showed that Kaiso-depleted cells underwent mesenchymal-to-epithelial transition and exhibited a more epithelial phenotype (that is, increased E-cadherin and ZO-1 but decreased Slug, ZEB1 and Vimentin expression), the effect of Kaiso depletion on the metastatic potential of breast tumor cells may be partially attributed to the attenuated EMT phenotype observed in these cells.

EMT is itself regulated by several distinct signaling pathways.^35^ Thus, it was intriguing to find that Kaiso expression positively correlates with the expression of several members of the TGFβ signalsome. Importantly, Kaiso associates with proximal TGFβR1 and TGFβR2 promoter regions, and Kaiso depletion results in reduced TGFβR1 and TGFβR2 expression, and attenuated TGFβ signaling. Consequently, TGFβ-dependent activation of target genes like *ANGPTL4* and *ZEB1* that are known to promote tumor dissemination and invasiveness^18, 46^ was impaired by Kaiso silencing. As the TGFβ pathway is highly implicated in BCa metastasis, the effect of Kaiso depletion on the metastasis of MDA-231 and Hs578T cells may be due to attenuation of TGFβ signaling in these cells, that is, loss of Kaiso-dependent regulation of TGFβR1/2 expression.

Several studies suggest that expression levels of the TGFβ receptors (high vs low) may determine the biological specificity of the TGFβ signaling cascade and the differential activation of Smad vs non-Smad signaling pathways.^15, 16, 17^ Our finding that Kaiso regulates expression of both TGFβR1 and TGFβR2 raises the possibility that Kaiso plays a central role in TGFβ-mediated tumorigenic effects. Consistent with this theory, our studies revealed that high Kaiso and TGFβR1 but not TGFβR2 expression is associated with poor overall survival in BCa patients. As metastasis accounts for poor overall survival in cancer patients, we surmise that Kaiso-dependent regulation of TGFβR1 but not TGFβR2 promotes TNBC metastasis.

**Figure 8:**
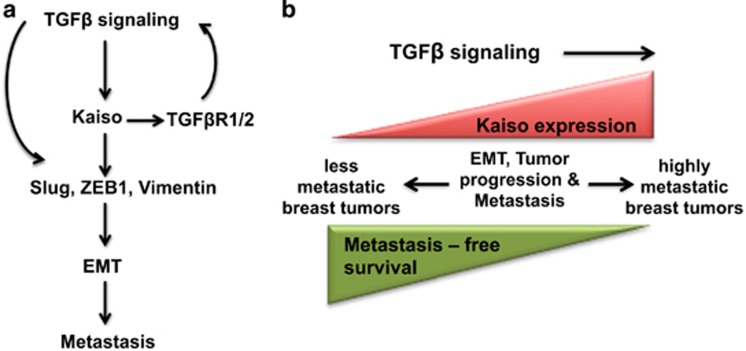
Potential model of the role of Kaiso in tumor progression and metastasis. (**a**) TGFβ signaling increases Kaiso expression that in turn promotes TGFβ signaling through increased expression of TGFβR1 and/or TGFβR2. TGFβ and Kaiso then promote EMT through increased expression of Slug, ZEB1 and/or Vimentin. (**b**) Less aggressive breast cancers exhibit low Kaiso expression, whereas highly metastatic breast tumors display high Kaiso expression, correlating with shorter metastasis-free survival. However, it remains to be determined whether high Kaiso expression occurs before tumor cells become highly aggressive or vice versa.

Our unexpected finding that TGFβ treatment increased Kaiso expression in breast tumor cells suggests that TGFβ signaling may positively regulate Kaiso expression, and thus form a positive feedback loop that enhances TGFβ-mediated signaling and metastasis (Figure 8a). Intriguingly, Kaiso may itself be required for TGFβ signaling or participate in other pathways implicated in BCa metastasis as overexpression of a kinase-active TGFβR1 in Kaiso-depleted MDA-231 cells was insufficient to rescue their metastatic abilities. Such findings are consistent with our model (Figure 8b), and other studies that have implicated increased Kaiso expression in the aggressiveness and overall survival of BCa patients.^20, 29^ However, it remains to be determined whether increased TGFβ signaling first induces high Kaiso expression or vice versa.

Collectively, these data implicate Kaiso as an important factor in TNBC aggressiveness and metastasis and suggest that it may be a relevant target for the development of therapies that will restrain the metastasis of aggressive breast cancers such as those of the TNBC subtype. Our finding that Kaiso can modulate TGFβ signaling further suggests that targeting Kaiso will alter the pro-metastatic phenotype associated with TGFβ signaling in advanced breast cancers.

## Materials and methods

### Cell culture

The human breast cancer cell lines MDA-231 and MCF-7 were obtained from ATCC (Manassas, VA, USA), and Hs578T were a generous gift from Dr John Hassell (McMaster University, Hamilton, Canada). All cell lines were cultured as previously described.^47^ For all TGFβ treatments, 10 ng/ml of TGFβ1 (R&D Systems, Minneapolis, MN, USA) was used.

### Generation of stable Kaiso-depleted cell lines

Kaiso depletion was achieved using a pRetroSuper (pRS) vector containing Kaiso-specific shRNAs (sh-Kaiso) that targeted the mRNA sequences, 5′-AAAAGATCATTGTTACCGATT-3′ referred to as sh-K1, or 5′-TTTTAACATTCATTCTTGGGAGAAG-3′ referred to as sh-K2. Then, 6 μg of pRS-sh-Kaiso plasmid or control vector (pRS-Kaiso scrambled) were transfected into MDA-231 or Hs578T using the Turbofect transfection reagent (Thermo Scientific, Waltham, MA, USA) according to the manufacturer's instructions. At 48 h post transfection, cells were treated with Puromycin (Invitrogen, Carlsbad, CA, USA) at 0.8 μg/ml (MDA-231) or 1.5 μg/ml (Hs578T) to select for stable Kaiso knockdown. Optimal Kaiso depletion was confirmed using immunoblot analysis of whole-cell lysates of individual clones, and clones exhibiting efficient Kaiso knockdown were selected for further studies.

### Immunoblot analysis

Immunoblot analysis was performed as previously described.^48^ Primary antibody incubations were performed overnight at 4 °C at the following dilutions: rabbit anti-Kaiso polyclonal (1:10 000); mouse anti-E-cadherin monoclonal (BD Biosciences, Mississauga, ON, Canada 610182; 1:1000); rabbit anti-ZO-1 polyclonal (Invitrogen 40-2200; 1:4000); rabbit anti-Vimentin polyclonal (Cell Signalling Technology (CST, Danvers, MA, USA) D21H3-XP); 1:1000); rabbit anti-ZEB1 polyclonal (Santa Cruz Technology, Dallas, TX, USA, H-102; 1:1000); rabbit anti-Slug polyclonal (CST-C19G7; 1:1000); rabbit anti-TGFβR1 monoclonal (CST-3712; 1:1000); rabbit anti-TGFβR2 polyclonal (Santa Cruz Technology sc-400; 1:2000); rabbit anti-phospho Smad2 monoclonal (Ser465/467) (CST-D43B4-XP; 1:800); rabbit anti-Smad2/3 monoclonal (CST-3102; 1:1000); mouse anti-β-actin monoclonal (Sigma Aldrich, Oakville, ON, Canada; 1:50 000). Primary antibody signals were amplified with horseradish peroxidase-conjugated goat anti-rabbit or donkey anti-mouse secondary antibody (Jackson ImmunoResearch Laboratories, West Grove, PA, USA) in a 1:10 000 dilution before visualization using Clarity Western Enhanced Chemiluminescence Substrate and the Bio-Rad ChemiDoc MP imaging system (Bio-Rad Laboratories, Mississauga, ON, Canada). All immunoblot experiments were performed in triplicate.

### Rescue experiments

A pCDNA3 vector expressing the sequence encoding the murine Kaiso cDNA (mKaiso) that is not targeted by the Kaiso-specific shRNA was utilized for Kaiso rescue experiments. Transient transfection of the pCDNA3 mKaiso vector into MDA-231-sh-K1 was achieved using Turbofect. At 3 weeks post transfection, whole-cell lysates obtained from the pCDNA3-mKaiso and pCDNA3-empty (control) transfected cells were subjected to immunoblot analysis of specified proteins after transient selection in media containing 0.8 μg Puromycin and 1000 μg Geneticin. For rescue of TGFβ signaling, constitutively active TGFβR1 (TGFβR1^T204D^), hereafter TβRI^204D^, was stably transfected into Kaiso-depleted MDA-231 cells using Turbofect. At 48 h post transfection, cells were treated with selection media containing 1000 μg Geneticin and Puromycin (Invitrogen) at 0.8 μg/ml to select for stable TRI^204D^-overexpressing Kaiso-depleted clones. Total protein isolated from control and experimental (TβRI^204D^) Kaiso-depleted cells was used for immunoblot analysis. Where applicable, all experiments were performed in triplicate.

### Reverse transcription–PCR (RT–PCR)

RNA was isolated from control and Kaiso-depleted breast cancer cells using the RNeasy mini kit (Qiagen, Hilden, Germany). cDNA synthesis and RT–PCR analysis were performed using the Superscript One-Step RT–PCR with Platinum Taq kit (Invitrogen) and the primers are indicated in Table 3. RT–PCR reactions were performed using the Eppendorf–Thermal cycler (Eppendorf, Hauppauge, NY, USA) under the following conditions: reverse transcription at 50 °C for 30 min, followed by initial denaturation at 95 °C for 5 min, 30 cycles of denaturation at 95 °C for 30 s, annealing at the specified temperature as indicated in Table 3 for 30 s, extension at 72 °C for 30 s, followed by a final extension at 72 °C for 10 min. Then, 10 μl of each RT–PCR reaction was electrophoresed on 1% agarose/ethidium bromide gels and images captured using the Bio-Rad ChemiDoc MP imaging system. All experiments were performed in triplicate.

### Quantitative RT–PCR

RNA (1 μg) isolated using the GeneJet RNA-plus isolation kit (Macherey-Negel) from control and TGFβ1-treated Kaiso-depleted cells was converted to cDNA using the qScript cDNA SuperMix (Quanta BioSciences, Gaithersburg, MD, USA) according to the manufacturer's protocols. For quantitative RT–PCR reactions, cDNA was amplified using the PerfeCta SYBR Green SuperMix ROX (Quanta BioSciences) as described in Pierre *et al.*,^43^ with the primers indicated in Table 4. The expression of each target was determined using a standard curve and normalized to the expression levels of β-actin. Statistical significance (using *t*-test and one-way analysis of variance with Tukey's test where appropriate) was determined using data obtained from at least three trials.

### Electrophoretic mobility shift assay

Double-stranded oligonucleotides corresponding to the specified KBS in the TGFβR1 and TGFβR2 promoters (see Table 1) were biotin-labeled using a Biotin 3′ End DNA Labeling kit (Pierce Biotechnology, Rockford, IL, USA) as per the manufacturer's protocol. TGFβR1 probes containing a CpG dinucleotide (KBS2–4) were also methylated with the CpG methyltransferase (M.SssI; New England Biolabs, Ipswich, MA, USA) as described in Pierre *et al.*^49^ Following biotinylation, complementary oligonucleotides were annealed by heating to 90 °C for 1 min and then allowed to cool slowly to room temperature. The reaction was then frozen and stored at −20 °C until use. Binding reactions were performed using 100 fmol of biotinylated double-stranded DNA probe and 200 ng of purified protein in 20 μl of binding buffer (10 mM Tris pH 7.5, 100 mM NaCl, 1 mM EDTA, 25% Glycerol, 1 mM dithiothreitol and Halt protease phosphatase inhibitor cocktail). To eliminate nonspecific binding, reaction mixtures were first incubated with 2 μg poly (deoxyinosinic-deoxycytidylic) acid (poly dI-dC) on ice for 1 h. Reaction mixtures containing biotinylated double-stranded DNA probe were incubated at room temperature for 30 min. For competition assays, a 100-fold excess (10 pmol) of unlabeled (cold) DNA was added. Reaction mixtures were loaded onto a 4.8% non-denaturing polyacrylamide gel and electrophoresed in 0.5 × TBE at 100 V at 4 °C. Nucleic acids were transferred onto a nylon membrane in 0.5 × TBE and the membrane crosslinked using a 312 nm UV lamp for 10 min. Visualization was performed utilizing a horseradish peroxidase-conjugated streptavidin Chemiluminescent Nucleic Acid Detection Module kit (Pierce) and hyperfilm (GE Healthcare, Mississauga, ON, Canada, 28906839) according to the manufacturer's protocol. Where applicable, experiments were performed in triplicate.

### ChIP and ChIP–PCR

MDA-231 and Hs578T cells were cultured to achieve ~80% confluency before chromatin isolation. Treatment with the demethylating agent, 5-azacytidine, ChIP and ChIP–PCR experiments were performed as previously described.^42, 49^ The following primers (−1035/−1008) KBS forward: 5′-AGGGCAAATTGGGACTGGAG-3′ and (−1035/−1008) KBS reverse: 5′-GAGGCCTGCAACTTGCTCTA-3′ at 65 °C, (−35/−29) KBS forward: 5′-CAGCTGAAAGTCGGCCAAAG-3′ and (−35/−29) KBS reverse: 5′-AGCCCCTAGCTCTCTCGTAG-3′ were used to amplify minimal TGFβR1 and TGFβR2 promoter regions respectively containing one or more core KBS (CTGCnA) and/or CpGs. The following primers were used as negative controls to confirm Kaiso binding specificity: (−2960/−2725) TGFβR1 negative-forward: 5′-GGAGCCTGGGAAATTGACAT-3′ and (−2960/−2725) TGFβR1 negative-reverse: 5′-CTCCAGTGCCTTGTACCCTG-3′, and (−2642/−2274) TGFβR2 negative-forward: 5′-TTGCCCAAGTTCCTCCAGAT-3′ and (−2642/−2274-) TGFβR2 negative-reverse: 5′-TTGCCCAAGTTCCTCCAGAT-3′.

### Ethics statement and metastasis studies

All experiments with NOD.Cg-*Prkdc*^scid^ *I/2rg*^tm1Wjl/^SzJ or NOD SCID Gamma (NSG) mice were approved by the Animal Research Ethics Board at McMaster University (AUP Number 14-05-14) and performed in accordance with the guidelines of the Animal Research Ethics Board. Female and male NSG mice were purchased from Charles River (Wilmington, MA, USA). To study Kaiso depletion effects on *in vivo* breast tumor metastasis, we injected 4.5 × 10^6^ Kaiso-shRNA or control-shRNA MDA-231 or Hs578T breast tumor cells in a Dulbecco's modified Eagle's medium/serum-free media/Matrigel mixture under the fourth mammary fat pad of the right abdominal mammary gland of ~5–8-week-old female NSG mice. No randomization was used in our studies as we used similar-aged pups obtained from the same breeding pair for each experiment. Most experiments were performed using at least five mice/treatment condition. Non-invasive monitoring of mice was performed weekly, and increased to 2–3 times weekly upon tumor appearance. Tumor growth was monitored externally with vernier calipers and tumor volume (in mm^3^) measured using the following formula (length/2 × width^2^) 2–3 times weekly. Mice were killed when tumor volume reached end point (~3300 mm^3^), and necropsies performed blindly by a veterinary pathologist to detect macrometastases. Tissues were perfused and fixed in 10% formalin before harvest and histological examination.

### Experimental metastasis studies

For experimental metastasis, 5 × 10^5^ MDA-231 and 1 × 10^6^ Hs578T control (Ctrl) and sh-Kaiso (sh-K) cells resuspended in 200 μl 1 × phosphate-buffered saline (PBS) were injected into the tail veins of ~6-week-old female NSG mice, whereas 1 × 10^6^ MDA-231 Ctrl, sh-Kaiso empty (sh-K-E) and TGFβR1^204D^ (sh-K-TR1^204D^) cells were injected into ~6-week-old female NSG mice (*n*=5/cell line). Mice were killed 5–6 weeks post injection and harvested tissues embedded in paraffin before the preparation of 5 μM thick tissue sections on slides that were subsequently H&E stained.

### Immunohistochemical staining of xenograft tissues

Harvested xenografts were embedded in paraffin before the preparation of 5 μM thick tissue sections on slides that were either stained with H&E, mouse anti-Kaiso 12H monoclonal (1:800)^50^ or p-Smad2 (CST-138D4; 1:200 for MDA-231 xenografts and 1:50 for Hs578T xenografts) primary antibodies overnight at 4 °C. Briefly, xenograft tissues were dewaxed by warming on a slide warmer at 60 °C for 20 min followed by immersion in xylenes 3 × 5 min. All other steps were performed as previously described,^31^ but we utilized PBS in place of TBS. Images were obtained using the Aperio Slide scanner (Leica Biosystems, Concord, ON, Canada).

### Gene expression analysis of TCGA and GEO data sets

Level 3 IlluminaHiSeq_RNASeqV2 expression (Illumina, iNC., San Diego, CA, USA) and associated clinical data were downloaded for all available patients from the TCGA data portal^51^ (19 March 2014; *n*=977). We used RSEM-quantified gene expression values to represent gene expression.^52^ For consistency, we used transcript levels of the genes *ESR1* and *ERBB2* to assign estrogen receptor and HER2 status to each patient. Transcript profiling data from the GEO dataset, GSE20685 (*n*=327), was performed on Affymetrix U133 Plus 2.0 gene chips (Affymetrix, Santa Clara, CA, USA) and downloaded from the GEO website.^35^ Robust Multi-Array was used to pre-process the dataset and gene expression values were calculated based on median expression of all probe sets mapping to a given gene based on Unigene ID. All genomic data processing was completed using R software.

### Statistical analysis

All statistical tests were completed using GraphPad Prism statistical software (GraphPad Software, Inc., La Jolla, CA, USA), and *P*<0.05 indicated significance. Data are presented as means±s.e.m. Unpaired Student's *t*-test was used for statistical analysis of two data sets, whereas one-way analysis of variance with Tukey/Newman–Keuls test was used for analysis of more than two data sets.

## Figures and Tables

**Table 1:** Oligonucleotides representing different potential Kaiso-binding sites in the TGFβR1 promoter

*TGFβR1 probe name*	*Oligonucleotide sequence (5*′*–3*′)	*Location*
KBS-1 WT	CTGAT**TCCTGCTA**TCAAGGTTTA	−1212 to −1208
KBS-1 MUT	CTGAT**TCCT***T***CTA**TCAAGGTTTA	
KBS-2 WT	ATTTTGGCG**T**CG**CAG**AGGGAAGGTGGGTGGAGCGTC**T**CG**CAG**TAAATTAG	−1035 to −1002
KBS-2 MUT	ATTTTGGCGT**C***T***CAG**AGGGAAGGTGGGTGGAGCGTC**TC***T***CAG**TAAATTAG	
KBS-3 WT	GGTGCTGGGGC**TGGCAG**ACCCCGCC	−289 to −283
KBS-3 MUT	GGTGCTGGGGC*G***GGCAG**ACCCCGCC	
KBS-4 WT	GCTGGGTCCCGCT**TGGCAG**CTCG	−117 to −111
KBS-4 MUT	GCTGGGTCCCGCT*G***GGCAG**CTCG	

Abbreviations: KBS, Kaiso-binding sequence; MUT, mutated; TGFβR1, transforming growth factor β receptor 1; WT, wild type. KBS—emboldened; CpG dinucleotides—underline; mutated nucleotides—italic.

**Table 2:** Oligonucleotides representing different potential Kaiso-binding sites in the TGFβR2 promoter

*TGFβR2 probe name*	*Oligonucleotide sequence (5*′*–3*′)	*Location*
KBS-1 WT	ATGGGCTGG**TGGCAG**AAGAGGGA	−1401 to −1395
KBS-1 MUT	ATGGGCTGG**TGG***A***AG**AAGAGGGA	
KBS-2 WT	CCCTTGCCT**CTGCAA**TCTTCCTC	−1081 to −1075
KBS-2 MUT	CCCTTGCCT**CT***T***CAA**TCTTCCTCT	
KBS-3 WT	TTACAGTTT**CTGCTA**TACTCTATA	−707 to −701
KBS-3 MUT	TTACAGTTT**CTG***A***TA**TACTCTATA	
KBS-4 WT	AAACATGAT**TGGCAG**CTACGAGA	−35 to −29
KBS-4 MUT	AAACATGAT**TGG***A***AG**CTACGAGA	

Abbreviations: KBS, Kaiso-binding sequence; MUT, mutated; TGFβR2, transforming growth factor β receptor 2; WT, wild type. KBS—emboldened; mutated nucleotides—italic.

**Table 3:** List of primer sequences used for RT–PCR analysis with their annealing temperatures

*Target*		*Sequence (5'–3')*	*Annealing temperature*
Kaiso	Forward	TGCCTATTATAACAGAGTCTTT	55 °C
	Reverse	AGTAGGTGTGATATTTGTTAAAG	
E-cadherin	Forward	CACCCTGGCTTTGACGCCGA	63 °C
	Reverse	AAACGGAGGCCTGATGGGGCG	
ZO-1	Forward	CGGGAAGTTACGTGGCGAA	60 °C
	Reverse	CTCCATTGCTGTGCTCTTGG	
Vimentin	Forward	TACGTGACTACGTCCACCCG	63 °C
	Reverse	ATCTCCTCCTGCAATTTCTCCC	
Slug	Forward	AGACCCCCATGCCATTGAAG	63 °C
	Reverse	CTTCTCCCCCGTGTGAGTTC	
ZEB-1	Forward	AGAATTCACAGTGGAGAGAAGCC	53 °C
	Reverse	CGTTTCTTGCAGTTTGGGCATT	
β-Actin	Forward	CTCTTCCAGCCTTCCTTCCT	55 °C

Abbreviation: RT–PCR, reverse transcription–PCR.

**Table 4:** List of primer sequences used for qRT–PCR analysis with their annealing temperatures

*Target*	*Sequence (5'–3')*		*Annealing temperature*
Kaiso	Forward	AGAGGAAAGGGCATGGAGAGT	60.8 °C
	Reverse	GGCCACGTTGCTCATTCAAG	
TGFβR1	Forward	CCGTTTGTATGTGCACCCTC	60 °C
	Reverse	GCCAGGTGATGACTTTACAGTAGT	
TGFβR2	Forward	CTCGGTCTATGACGAGCAGC	60 °C
	Reverse	CCTCCATTTCCACATCCGACT	
ZEB-1	Forward	AGAATTCACAGTGGAGAGAAGCC	60 °C
	Reverse	CGTTTCTTGCAGTTTGGGCATT	
ANGPTL4	Forward	CAGCCTGCAGACACAACTCA	60 °C
	Reverse	ATTCGCAGGTGCTGCTTCTC	
β-Actin	Forward	CTCTTCCAGCCTTCCTTCCT	55 °C
	Reverse	AGCACTGTGTTGGCGTACAG	

Abbreviation: qRT–PCR, quantitative reverse transcription–PCR.
