# Supplementary figures and images for: Kaiso depletion attenuates transforming growth factor-β signaling and metastatic activity of triple-negative breast cancer cells
Source: Oncogenesis. 2016 Mar 21;5(3):e208–. doi: 10.1038/oncsis.2016.17 (PMC4815049; doi:10.1038/oncsis.2016.17)

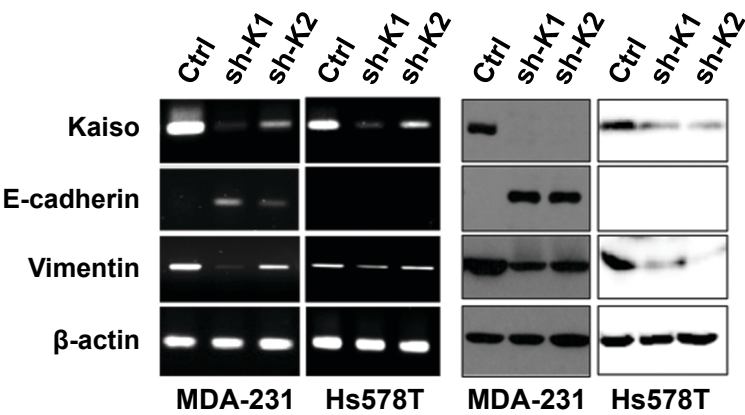

Supplement: Supplementary Figure 1 [file oncsis201617x1.pdf]

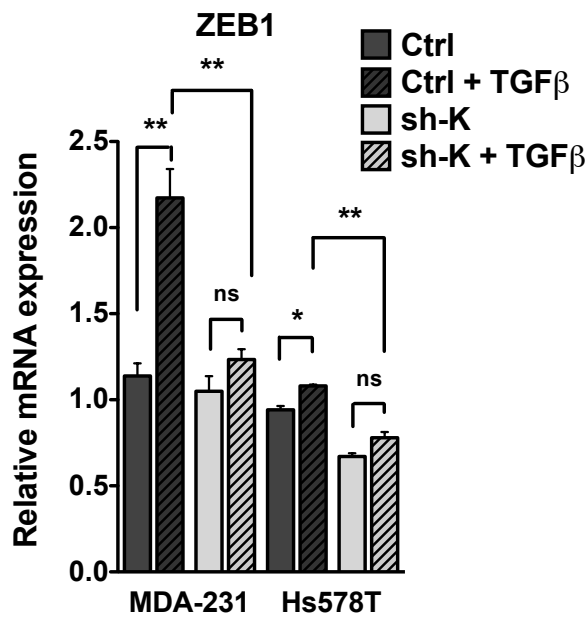

Supplement: Supplementary Figure 2 [file oncsis201617x2.pdf]

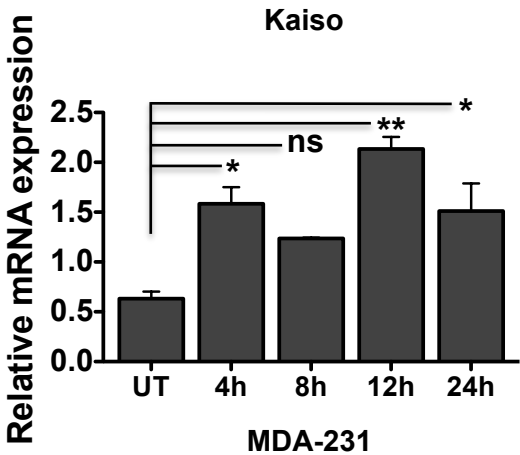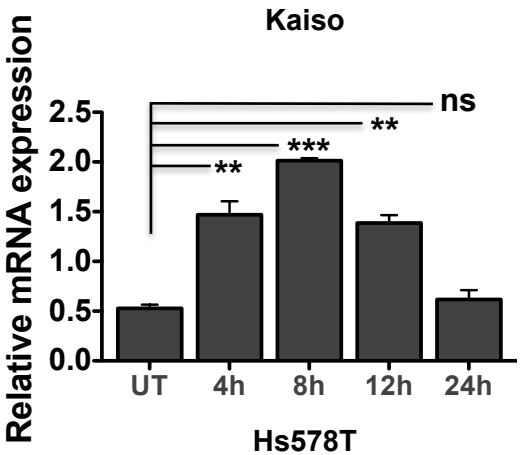

Supplement: Supplementary Figure 3 [file oncsis201617x3.pdf]

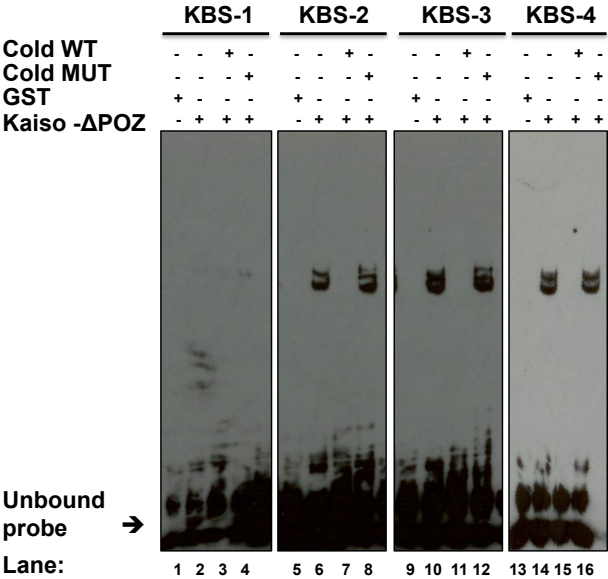

Supplement: Supplementary Figure 4 [file oncsis201617x4.pdf]

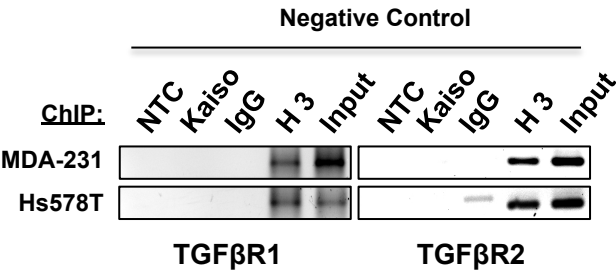

Supplement: Supplementary Figure 5 [file oncsis201617x5.pdf]

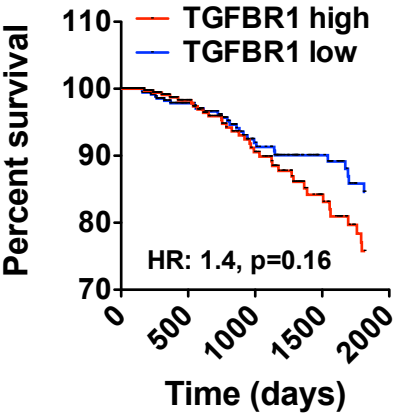

Supplement: Supplementary Figure 6 [file oncsis201617x6.pdf]
