# Supplementary material for: Kaiso depletion attenuates transforming growth factor-β signaling and metastatic activity of triple-negative breast cancer cells
Source: Oncogenesis. 2016 Mar 21;5(3):e208–. doi: 10.1038/oncsis.2016.17 (PMC4815049; doi:10.1038/oncsis.2016.17)
Supplement: Supplementary Figure Legends [file oncsis201617x7.pdf]

Supplemental Figure Legends

Supp. Figure 1: ***Kaiso depletion attenuates the expression of several EMT-related proteins and induces MET.*** Kaiso was efficiently depleted by a Kaiso-specific shRNA targeting two different regions of the Kaiso mRNA (sh-K-1 and sh-K-2). Kaiso depletion increased expression of E-cadherin in MDA-231, and decreased expression of Vimentin in both MDA-231 and Hs578T cells transfected with either sh-K-1 or sh-K-2 as assessed by RT-PCR and immunoblot analysis.

Supp. Figure 2: ***Kaiso depletion attenuates TGFβ–induced expression of ZEB1.*** TGFβ-induced expression of *ZEB1* is attenuated in Kaiso-depleted cells treated with 10ng/mL of TGFβ for 24 hours. * = $p < 0.05$, ** = $p <0.005$.

Supp. Figure 3: ***Kaiso-expression is increased in response to TGFβ signaling.*** qRT-PCR analysis indicating the expression of Kaiso transcripts at different time points in response to TGFβ treatment. One-way ANOVA with Newman-Keuls test was used for statistical analysis. * = $p < 0.05$, ** = $p <0.005$, *** = $p <0.0001$.

Supp. Figure 4: ***Kaiso specifically associate with the TGFβR2 promoter.*** EMSA analysis showing the specificity of Kaiso binding to the TGFβR2 promoter. Unlabeled cold mutated (MUT) probes is incapable of competing with labeled wildtype (WT) probes (lanes 8, 12, 16) compared with unlabeled cold WT probes which competes for

Kaiso binding (lanes 7, 11, 15). m – methylated. Representative images shown. All experiments were conducted in triplicate.

Supp. Figure 5: ***Kaiso specifically interacts with the TGFβR1 and TGFβR2 promoter in vivo.*** ChIP experiments of MDA-231 and Hs578T chromatin showing that Kaiso does not bind a region upstream of the TGFβR1 and TGFβR2 promoter *in vivo* lacking the presence of CpGs or Kaiso binding sequences. NTC – no template control. H3 – Histone3 positive control. Input – 10% input.

Supp. Figure 6: ***High TGFβR1 expression does not correlate with poor prognosis in breast cancer patients.*** Kaplan-Meier survival curves showing that high TGFβR1 expression does not correlate significantly with overall survival in the TCGA breast cancer dataset
